# Exploration of predictive and prognostic alternative splicing signatures in lung adenocarcinoma using machine learning methods

**DOI:** 10.1186/s12967-020-02635-y

**Published:** 2020-12-07

**Authors:** Qidong Cai, Boxue He, Pengfei Zhang, Zhenyu Zhao, Xiong Peng, Yuqian Zhang, Hui Xie, Xiang Wang

**Affiliations:** 1 Department of Thoracic Surgery, The Second Xiangya Hospital, Central South University, Changsha, 410011 Hunan China; 2 Hunan Key Laboratory of Early Diagnosis and Precision Therapy, Department of Thoracic Surgery, The Second Xiangya Hospital, Central South University, Changsha, 410011 China

**Keywords:** Lung adenocarcinoma, Alternative splicing, Random forests, Splicing switch, Metastasis, Prognosis

## Abstract

**Background:**

Alternative splicing (AS) plays critical roles in generating protein diversity and complexity. Dysregulation of AS underlies the initiation and progression of tumors. Machine learning approaches have emerged as efficient tools to identify promising biomarkers. It is meaningful to explore pivotal AS events (ASEs) to deepen understanding and improve prognostic assessments of lung adenocarcinoma (LUAD) via machine learning algorithms.

**Method:**

RNA sequencing data and AS data were extracted from The Cancer Genome Atlas (TCGA) database and TCGA SpliceSeq database. Using several machine learning methods, we identified 24 pairs of LUAD-related ASEs implicated in splicing switches and a random forest-based classifiers for identifying lymph node metastasis (LNM) consisting of 12 ASEs. Furthermore, we identified key prognosis-related ASEs and established a 16-ASE-based prognostic model to predict overall survival for LUAD patients using Cox regression model, random survival forest analysis, and forward selection model. Bioinformatics analyses were also applied to identify underlying mechanisms and associated upstream splicing factors (SFs).

**Results:**

Each pair of ASEs was spliced from the same parent gene, and exhibited perfect inverse intrapair correlation (correlation coefficient = − 1). The 12-ASE-based classifier showed robust ability to evaluate LNM status of LUAD patients with the area under the receiver operating characteristic (ROC) curve (AUC) more than 0.7 in fivefold cross-validation. The prognostic model performed well at 1, 3, 5, and 10 years in both the training cohort and internal test cohort. Univariate and multivariate Cox regression indicated the prognostic model could be used as an independent prognostic factor for patients with LUAD. Further analysis revealed correlations between the prognostic model and American Joint Committee on Cancer stage, T stage, N stage, and living status. The splicing network constructed of survival-related SFs and ASEs depicts regulatory relationships between them.

**Conclusion:**

In summary, our study provides insight into LUAD researches and managements based on these AS biomarkers.

## Background

Lung cancer is the most common and deadliest cancer worldwide, in which non-small cell lung cancer (NSCLC) accounts for 85% of all cases [1, 2]. NSCLC can be mainly classified into lung adenocarcinoma (LUAD), squamous cell carcinoma, and large cell carcinoma, among which LUAD is the major histological subtype. Although scientists and clinicians around the world have been making great efforts in the fight against LUAD, the survival outcome of LUAD is still poor because of the complexity of tumor initiation and progression, with an average 5-year survival rate of 15% [3]. Therefore, intensive study to provide more effective diagnostic and treatment strategies for patients with LUAD is of particular importance.

Findings of The Human Genome Project indicated a truth that the number of human protein-coding genes (less than 25,000) is far less than the previous estimation from the diversity of human proteome (including approximately 100,000 proteins). Further studies revealed this proteomic diversity may be attributed to post-transcriptional processing in the RNA level. Recent estimates indicated that nearly 95% of human genes are involved in alternative splicing (AS), where a pre-mRNA can be spliced into several mRNA isoforms with different functions [4]. Apart from increasing protein complexity, translation of mRNA isoforms can also be inhibited by AS through the introduction of a premature stop codon causing degradation [5]. The dysregulation of AS is implicated with multiple diseases. A growing amount of evidence showed that cancer cells exhibit massive aberrant splicings [6–8]. Many studies also demonstrated that the switching from oncogenic splicing isoforms to protective ones for certain genes represents crucial events in cancer [9]. These abnormal AS events (ASEs) consist of various tumorigenesis processes including cell proliferation, cell death inhibition, immune escape, and inducing angiogenesis [10, 11]. In addition, uncontrolled expression of splicing factors (SFs) promotes the emerging of numerous AS variants that drive carcinogenesis [12]. Recent studies targeting transcriptome and epigenetic alterations identified many molecules as promising diagnostic and therapeutic tumor biomarkers. Likewise, it is meaningful to integratively investigate the expression alterations of ASEs and identify tumor-specific ASEs for LUAD.

Machine learning is a discipline in computer science based on algorithms that parse data, learn from data, and make predictions or decisions on a wide variety of complex issues. The development of machine learning technology and its wide application in biomedical studies provide researchers with powerful tools to find the most informative detection markers from large, highly complex datasets [13]. In this study, we explored LUAD-related ASEs implicated in splicing switches, optimal AS signatures identifying lymph node metastasis (LNM) statuses of patients with LUAD, and a model to predict overall survival (OS) of patients with LUAD by applying machine learning algorithms to genome-wide AS data. Perfect inverse correlations (correlation coefficients = − 1) between the identified oncogenic isoforms and protective isoforms derived from the same genes were exhibited. Results also indicated the two signatures have robust predictive capacities. Random-forest based algorithm Boruta was used to evaluate the importance of ASEs for LUAD. Spearman correlation analysis was used to evaluate correlations among important ASEs which were originated from the same gene. Then a nested five-fold cross-validation algorithm was applied to decide the proper number of predictors in random forest classifiers. Cox regression model and random survival forest (RSF) algorithm were used to identify survival-related seed genes and the forward selection model was developed to identify prognosis-related key genes for model construction. Bioinformatical analyses were also performed to explore correlated pathways, identify upstream SFs, and analyze the correlations between the prognostic model and clinical variables.

## Methods

### Data collection and preprocessing

**Fig. 1:**
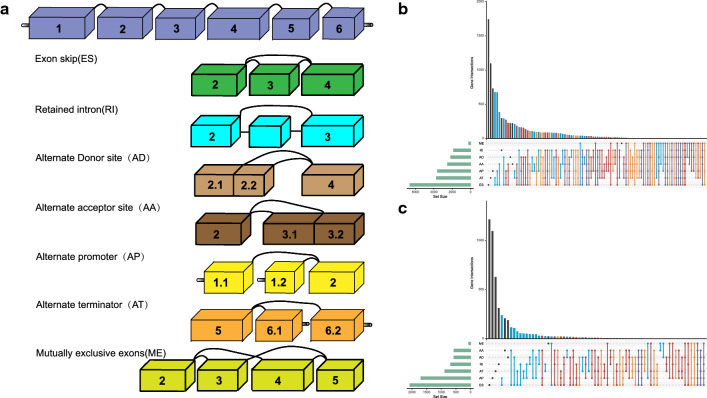
Illustrations of the seven AS types and UpSet plots. **a** Illustration of the seven types of AS. **b** Upset plot displaying the number of ASEs included in the current study in different types of splicing patterns. **c** Upset plot displaying selected ASEs after preliminary screening. *AS* alternative splicing, *SD* standard deviation, *ASE* alternative splicing event

The mRNA data in fragments per kilobase per million mapped reads format and patients’ clinical information of LUAD were retrieved from The Cancer Genome Atlas (TCGA) database (https://portal.gdc.cancer.gov/). AS data of LUAD were downloaded from TCGA SpliceSeq (https://bioinformatics.mdanderson.org/TCGASpliceSeq) [14]. The percent-spliced-in (PSI) value, representing the ratio between reads including or excluding exons, was calculated to describe detected ASEs. According to splicing patterns, all of these ASEs were classified into seven types: exon skip (ES), retained intron (RI), alternate donor site (AD), alternate acceptor site (AA), alternate promoter (AP), alternate terminator (AT), and mutually exclusive exons (ME) (Fig. 1a). Only ASEs available in more than 70% of samples were included in this study. Missing values were imputed using R package impute.

The name of each ASE consists of three parts: gene symbol, ID number designated in TCGA SpliceSeq database, and splicing type. For example, the name “CHEK-19309-AP” indicates the parent gene of this event is CHEK, its ID number in TCGA SpliceSeq database is 19309, and its splicing type is AP.

### Machine learning algorithms

#### Random forests

Random forests is an ensemble learning technique that makes a prediction based on constructing multiple unpruned decision trees, each of which is constructed on several bootstrap samples of the training set data using a subset of randomly picked variables [15]. The tree structure of random forests can be denoted as:

$$\{ h(X,\psi (t));t = 1, \ldots ,T\} .$$

In the formula, X is an input vector and ψ(t) represents the independent trees in random forests and each tree elects the most popular class for X via a unit vote. Then the decisions made by all the trees were aggregated and the class of X is determined based on the principle of majority voting. This supervised non-parametric machine learning method can help researchers acquire key information from massive complicated data and resist both overfitting and underfitting [16].

#### Boruta

Boruta algorithm is a random-forest based feature selection method. This algorithm estimates the importance of features and captures important features in the dataset [17]. Through the following workflow, the algorithm finds all features that have either strong or weak correlations with the outcome variable: (1) Boruta duplicates the given dataset and shuffles these added attributes to increase randomness. The new features are called shadow features. (2) It develops a random forest classifier on the extended dataset and gathers the importance of each feature, which was measured by Z-scores. Z-score is computed by dividing the average loss of mean decrease accuracy by its standard deviation (SD). The higher the Z-score, the more important the feature. (3) Then, the algorithm checks whether a real feature has a higher Z-score than the maximum Z-score among shadow attributes. If not, the real feature would be deemed as unimportant and removed. Afterward, another iteration would begin. (4) These procedures repeat until the importance of all the features is assigned or the algorithm reaches the preset limit of runs.

#### Random survival forests (RSF)

RSF is an extension of the original random forest technique which can be used for survival data [18]. Based on random forests, RSF splits decision trees on a predictor using the splitting criterion. A node of the decision tree is split on the predictor which makes differences across daughter nodes reaching the maximal. In this study, the differences were determined by a log-rank splitting rule. When the tree grows to its terminal node, the cumulative hazard function (CHF) for each node was calculated, which is calculated by the Nelson–Aalen estimator:

$$N_{b,h} (t) = \mathop \sum \limits_{{t_{l,h} \le t}} \frac{{d_{l,h} }}{{I_{l,h} }}.$$

In this formula, *h*, *b*, and *t* refer to the terminal node, survival tree, and time, respectively. *d*_*l,h*_ represents the number of death, *I*_*l,h*_ represents patients at risk, and *t*_*l,h*_ represents distinct time events. The same CHF is assigned to all cases in *h*. Then an ensemble CHF is computed for the survival forest with *B* trees for a given *d*-dimensional case *x*_*i*_:

$$H_{e}^{s} (t,x_{i} ) = \frac{1}{B}\mathop \sum \limits_{b = 1}^{B} \mathop \sum \limits_{h \in T\left( b \right)} H_{b,h}^{s} (t,x_{i} )$$

$H_{b,h}^{s} (t,x_{i} )$ in the above formula is calculated as [19]:

$$H_{b,h}^{s} \left( {t,x_{i} } \right) = \left\{ {\begin{array}{*{20}c} {N_{b,h} (t)} & {x_{i} \in h} \\ 0 & {otherwise.} \\ \end{array} } \right.$$

Through the above methods, RSF adapts traditional random forest algorithm and can handle problems associated with survival. And these procedures in the present study are carried by randomForestSRC R package. Using *Surv* and *var.select* functions in this package we preliminary screened out survival-related ASEs.

### Selection models

#### Cox regression model

Cox regression model is a model simultaneously analyzing the effects of several variables on survival. Based on the condition of the proportional hazard, this model assumes the hazard functions for different individuals are proportional and covariates’ effects on individuals are constant. Cox regression model can be formulated as:

$$h(t,X) = h_{0} (t ) {\text{exp}}\left( {\mathop \sum \limits_{i = 1}^{m} \beta_{i} X_{i} } \right).$$

In this formula, *h*_0_(*t*) is the baseline hazard function, t is a time variable, and β_i_ is a coefficient vector weighing the contribution of feature X_i_.

#### Forward selection model

We used a forward selection model to select prognosis-related genes from survival-related genes. This selection was achieved by rbsurv R package in the following procedures [20]: (1) The dataset was randomly divided into the training set (3/4 of all samples) and the validation set (1/4 of all samples). A gene was then fitted to the training set and the parameter estimate $\hat{\beta }_{i}^{0}$ for this gene was obtained. Next, $\hat{\beta }_{i}^{0}$ and the validation set were used to evaluate the log-likelihood. This process was repeated for each ASE. (2) The above procedures were repeated 100 times and we obtained 100 log-likelihoods for each ASE. Then the ASE with the largest mean log-likelihood was selected as the best ASE which is the most survival-associated one. Simultaneously, we selected the next best ASE by repeating previous procedures and found the optimal two-ASE model with the largest mean log-likelihood. (3) These forward selection methods continued until the fitting is impossible, resulting in a series of models. Then the Akaike Information Criterion (AIC) was calculated to evaluate these models to avoid overfitting. Finally, the model with the minimal AIC was selected as the final model.

### Workflow of the current study

#### Preliminary filtering

SD reflects the information entropy of a feature. The greater the SD, the more informative the feature. To filter out less informative ASE and to decrease the computation of subsequent analyses, we analyzed SDs of all the ASEs in the dataset and excluded ASEs with SD < 0.1. Besides, ASEs whose mean PSI ≤ 0.05 were also excluded.

#### Identification of LUAD-related ASEs implicated in splicing switch

First, to circumvent the problem caused by severely imbalanced data (10.3% normal, 89.7% LUAD) in the learning process, we balanced the proportions of normal and LUAD samples by oversampling normal samples using the *ovun.sample* function of ROSE R package. Thus, we generated augmented data with a balanced class distribution. Second, we applied Boruta algorithm to select ASEs which were important for distinguishing between normal and LUAD samples. Third, to further explore ASEs implicated in splicing switch, we separately analyzed the correlations of LUAD-related ASEs derived from the same gene.

#### Construction of classifier for recognizing LNM

Applying Boruta algorithm, we selected ASEs correlated with outcome variables (LNM or not). Using these ASEs, we performed nested five-fold cross-validation based on the random forest model. The cross-validation sequentially reduced the number of ASEs (ranked by variable importances from Boruta analysis) and this process repeated five rounds. The mean cross-validation error was calculated and the classifier with the minimum error rate was chosen. The classifier’s classification capacity was evaluated by cross-validation.

#### Model construction and functional enrichment analysis

Cox regression is the traditional method for survival analyses with an understandable output, while RSF provides more insight into the relative importance of model covariates [21]. Based on previous results, the combination of these two methods could produce results with higher confidence than a single one [21]. Therefore, RSF and Cox regression were performed for each ASE, and only ASEs which were survival-related in both methods were selected. Kyoto Encyclopedia of Genes and Genomes (KEGG) pathways analysis and Reactome pathways analysis were performed to analyze the functional categories of parent genes of survival-related ASEs using Cytoscape (version 3.7.2) plug-in ClueGO (version 2.5.5) [22, 23]. The dataset was further divided into the training set (3/4 of all samples) and the test set (1/4 of all samples). We used the forward selection model to identify prognosis-related ASEs and multivariate Cox regression to construct the prognosis model. The final model was tested in the internal test set. Relationships between the prognostic model and clinical-pathological variables were analyzed in the entire set using the Wilcoxon test. *P* < 0.05 was considered statistically significant.

#### Splicing network analysis

A total of 390 SFs were retrieved from SpliceAid 2 database (http://193.206.120.249/splicing_tissue.html) [24]. Univariate Cox analysis was used to identify survival-related SFs. *P *< 0.01 was considered to be significant. Spearman correlation analysis was conducted to evaluate the correlations between survival-related SFs and ASEs. The criterion of selecting correlated variables was *P *< 0.01 and |coefficient| > 0.2. Finally, their correlations were visualized via Cytoscape.

### Software

All statistical analyses were conducted using R software (version 3.6.3). R package Boruta, randomForest, RandomforestSRC, caret, survival, rbsurv, pROC, timeROC, pheatmap, and ggplot2 were used in this study for analyzing data or drawing purposes. All codes are available on GitHub (GitHub, Inc., San Francisco, California) at https://github.com/cqd1308/JTM-Rscript-ASsignatures. Cytoscape software was applied to conduct functional and pathway enrichment analysis and plot the network graph.

## Results

### Preparation of datasets

**Fig. 2:**
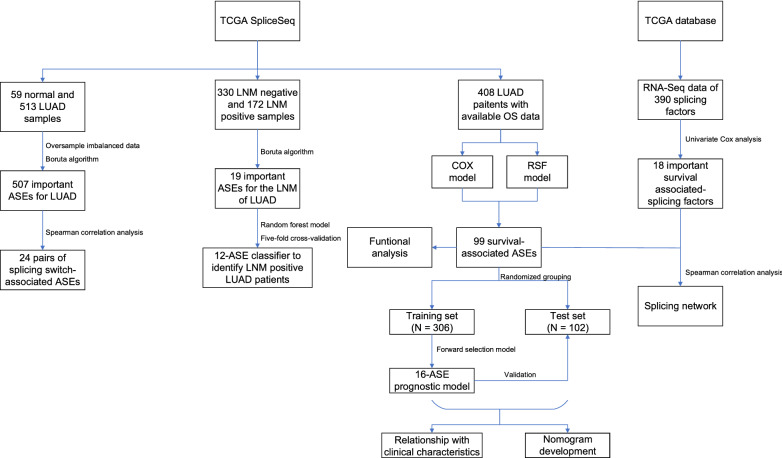
Flow chart of this study

The flow chart presenting the overall analysis process of the current study is shown in Fig. 2. After data preprocessing, 572 samples (59 normal, 513 LUAD) of LUAD-AS dataset were included in analyses for distinguishing between normal and LUAD samples. 502 samples (330 LNM negative, 172 LNM positive) of LUAD-AS dataset had available N stage data and were included for recognition of LNM. We removed samples with unavailable survival data and follow-up days less than 30, after which 408 samples were left for the prediction of OS. The 408 samples were randomly split into the training set and the test set consisting of 306 and 102 samples for model construction and evaluation, respectively. Besides, 380 overlapping samples in LUAD-mRNA dataset and LUAD-AS dataset were used in further regulatory SFs analysis. Detailed clinical information is shown in Additional file 1: Table S1 to Additional file 4: Table S4.

### Preliminary screening

A total of 43,948 ASEs corresponding to 10,367 genes were included in this study, of which ES had the highest proportion, which was 27.2%, and the lowest was contributed by ME, which had only 0.9%. One single gene could have seven splicing types at most (Fig. 1b). To exclude less discriminative features, we filtered out ASEs according to the criteria described in Methods section and got 10,951 ASEs (Fig. 1c) spliced from 4915 parent genes.

### LUAD-related ASEs implicated in splicing switch

**Fig. 3:**
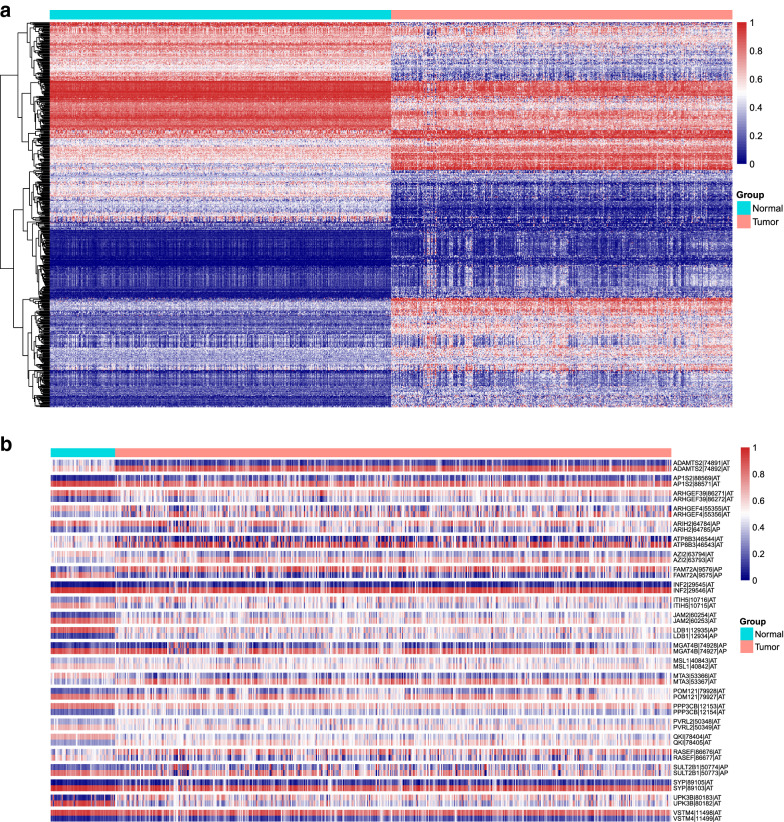
Identification of ASEs associated with splicing switches between normal and LUAD samples. **a** Heat map showing the PSI levels of important ASEs for differentiating between normal and LUAD samples after Boruta selection. **b** Heat map demonstrating PSI levels of the 24 pairs of ASEs implicated in splicing switches of LUAD development. Each pair of ASEs were perfectly contrarily expressed, and the expressions of these ASEs were distinct in normal and LUAD tissues

Using the oversampled dataset consisting of 513 normal and 513 LUAD samples, 506 ASEs from 360 parent genes were confirmed as important features for differentiating between LUAD and normal samples using Boruta feature selection (Fig. 3a), whose detailed data are summarized in Additional file 5: Table S5. Subsequently, we picked out and analyzed the correlations of ASEs derived from the same genes. Interestingly, as shown in Fig. 3b, intrapair correlation coefficients in 24 pairs of ASEs spliced from the same parent genes were − 1, indicating perfect negative correlations. This also suggests the splicing pattern shifts of these genes significantly contributed to the pro-oncogenic or anti-oncogenic transition.

### Classifier for LNM

**Fig. 4:**
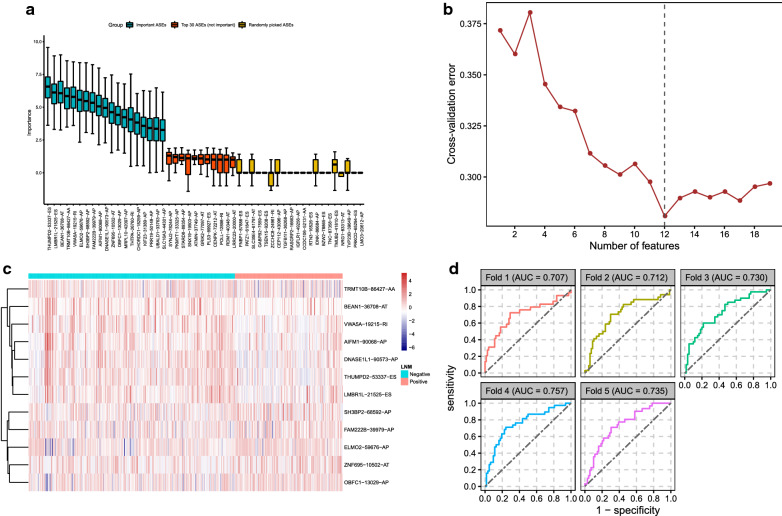
LNM classifier construction and the efficiency of the 12-ASE-based classifier. **a** Z-score of the top 30 important ASEs and 20 randomly picked ASEs using Boruta algorithm. **b** The mean cross-validation error of the five-round fivefold cross-validation about different numbers of ASEs. **c** The heat map showing PSI levels of ASEs in the LNM classifier. The data were normalized using R function *scale*. **d** ROC curves for the fivefold cross-validation of the classifier to identify LNM statuses of LUAD patients. *LNM* lymph node metastasis. Important ASEs, the ASEs confirmed as important features for the identification of LNM for LUAD patients by Boruta algorithm. Top 30 ASEs (rejected), the ASEs had the top 30 Z-score but rejected as unimportant features by Boruta algorithm for the identification of LNM for LUAD patients

Using Boruta feature selection, 19 ASEs spliced from 19 different genes were confirmed as important features for LNM (Additional file 6: Table S6). The Z-score of the top 30 ASEs with the highest Z-score and the other 20 randomly selected ASEs was shown in Fig. 4a. The 502 samples were randomly assigned to the training set or the test set by fivefold cross-validation. The 19 ASEs were removed from the random forest-classifier one by one from a lower Z-score to a higher Z-score, and each time a new ASE was removed, the classification performance was updated with the fivefold cross-validation. The cross-validation procedures were repeated five rounds and the average cross-validation error was calculated. The number of kept ASEs concerning the average error rate was shown in Fig. 4b and the best classification performance was achieved when the number of ASEs was 12. The 12 ASEs were: THUMPD2-53337-ES, LMBR1L-21525-ES, BEAN1-36708-AT, TRMT10B-86427-AA, VWA5A-19215-RI, ELMO2-59676-AP, SH3BP2-68592-AP, FAM222B-39979-AP, AIFM1-90068-AP, DNASE1L1-90573-AP, ZNF695-10502-AT, and OBFC1-13029-AP. The PSI value of kept features is shown in Fig. 4c. Results of ROC analysis (Fig. 4d) revealed the AUC values of the 12-ASE-based classifier were all more than 0.7 in fivefold cross-validation, indicating the robust sensitivity and specificity of this classifier for the recognition of LNM.

### Identification of survival-related ASEs and functional annotation

**Fig. 5:**
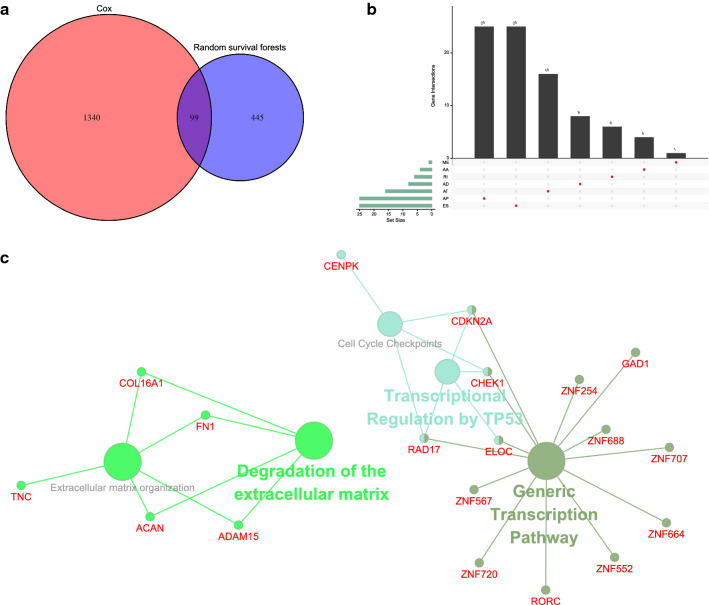
Identification of survival-related ASEs and pathway enrichment analyses. **a** Venn diagram summarizing survival-related ASEs identified by Cox regression and random survival forests. **b** Upset plot displaying overlapping ASEs between two methods. **c** Pathway analyses of genes associated with OS-related splicing events

Cox regression and RSF methods identified 1439 and 544 ASEs (Additional file 7: Table S7 and Additional file 8: Table S8), respectively. 99 ASEs were survival-related in both results (Fig. 5a). To explore underlying mechanisms of survival-related ASEs, 85 parent genes (Fig. 5b) of these ASEs were used for KEGG and Reactome pathway analyses. Enrichment results indicated pathways including “Transcriptional Regulation by TP53”, “Cell Cycle Checkpoints”, “Generic Transcription Pathway”, “Degradation of the extracellular matrix”, and “Extracellular matrix organization” were significantly enriched (Fig. 5c).

### Prognostic model for LUAD

**Fig. 6:**
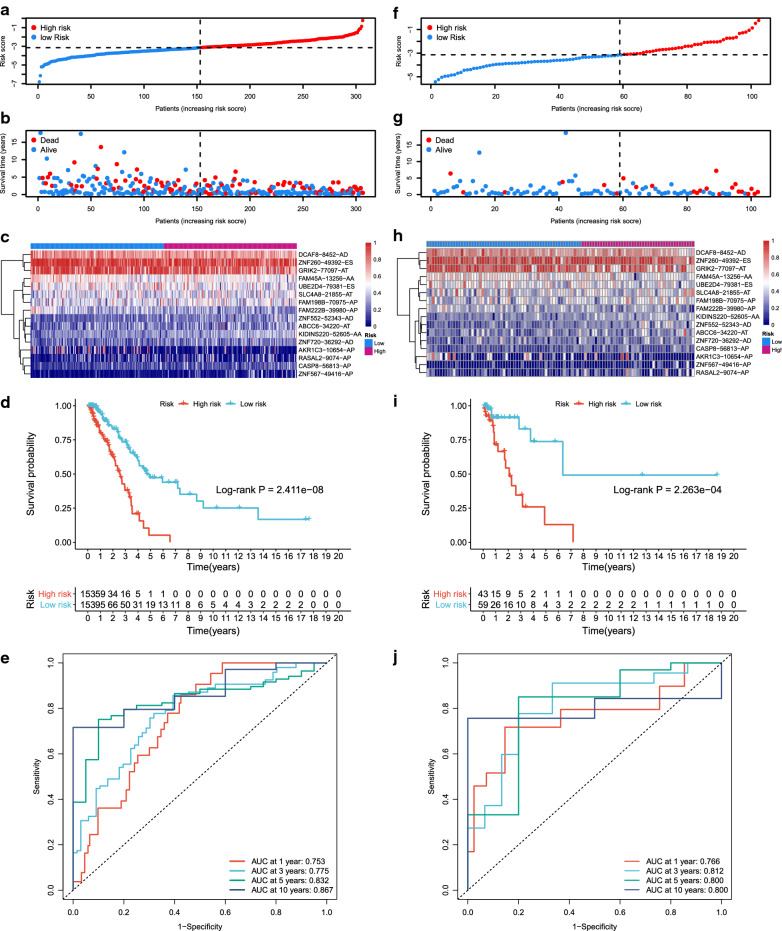
Prognostic model construction and efficiency assessment. **a**, **b** Visualization of the risk score and survival for each patient in the training set. **c** The heat map comparing the PSI levels of the 16-ASE signature in the high-risk and the low-risk group of the training set. **d** Kaplan–Meier survival curve for patients in the high-risk and the low-risk group of the training set. **e** Time-dependent ROC curves for LUAD patients in the training set. **f**, **g** Visualization of the risk score and survival for each patient in the test set. **h** The heat map comparing the PSI levels of the 16-ASE signature in the high-risk and the low-risk group of the test set. **i** Kaplan–Meier survival curve for patients in the high-risk and the low-risk group of the test set. **g** Time-dependent ROC curves for LUAD patients in the test set

Baseline characteristics of the training set and the internal test set were shown in Table 1, and no statistically significant difference in clinical features existed between the two sets. Then the 99 survival-associated ASEs were introduced to the forward selection model using R package rbsurv. Afterward, 16 key prognosis-related ASEs were selected and a prognostic risk score model for LUAD was established using multivariate Cox regression (Additional file 9: Table S9). Choosing the median risk score of the training set as the cut-off, samples were divided into the high-risk group and the low-risk group (Fig. 6a). As shown in Fig. 6b, the patients in the high-risk group had higher mortality than those in the low-risk group. The heat map showed the PSI levels of the 16 ASEs involved in the prognostic model (Fig. 6c), and the Kaplan–Meier curves showed a clear distinction between two risk groups (*P *< 0.001) (Fig. 6d). The AUC of this model in 1, 3, 5, and 10 years in the training set was 0.753, 0.775, 0.832, and 0.867, respectively (Fig. 6e). Samples in the internal test set were also divided into the high-risk group and the low-risk group according to the median risk score of the training set. The risk plot, the scatter diagram showing OS, and the heat map reflecting PSIs of key ASEs of the test set were shown in Fig. 6f–h. The Kaplan–Meier plot also showed a very significant difference (*P *< 0.001) between the high-risk group and low-risk group in the test set (Fig. 6i). ROC analysis revealed the robust predictive capacity of the 16-ASE-based model, where AUC in 1, 3, 5, and 10 years were 0.766, 0.812, 0.800, and 0.800, respectively (Fig. 6j).

**Table 1:** Baseline characteristics of the training set and the internal test

	Training set	Test set	P value
n	306	102	
Gender (%)			0.529
Female	166 (54.2)	51 (50.0)	
Male	140 (45.8)	51 (50.0)	
Age (median [IQR])	66.00 [59.00, 73.00]	68.00 [57.25, 72.75]	0.744
Vital status (%)			0.13
Alive	211 (69)	79 (77.5)	
Dead	95 (31)	23 (22.5)	
Smoking history (%)			0.287
Non-smoker	42 (14.1)	9 (9.3)	
Smoker	255 (85.9)	88 (90.7)	
Stage (%)			0.145
Stage I	161 (53.8)	54 (53.5)	
Stage II	68 (22.7)	25 (24.8)	
Stage III	58 (19.4)	13 (12.9)	
Stage IV	12 (4.0)	9 (8.9)	
OS_time (median [IQR])	367.50 [141.25, 937.25]	250 [108.00, 646.25]	0.054

*IQR* interquartile range, *OS* overall survival

**Fig. 7:**
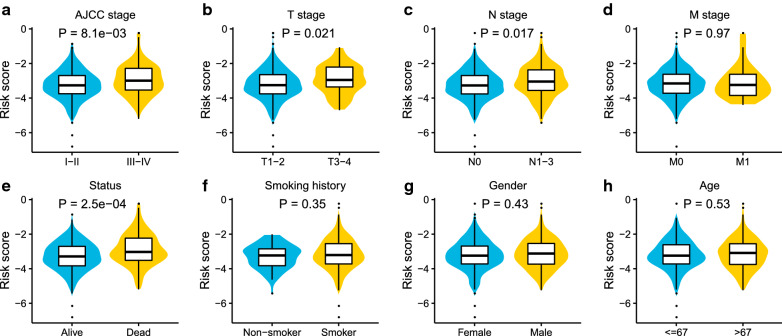
Relationships between clinical features and the risk model. The distribution of risk scores of LUAD patients in different clinical groups. LUAD patients were assigned to different groups according to clinical risk factors. **a** AJCC stage, **b** T stage, **c** N stage, **d** M stage, **e** vital status, **f** smoking history, **g** gender, **h** age

Further analysis of the risk score model indicated correlations between the 16-ASE prognostic model and clinical variables including American Joint Committee on Cancer (AJCC) stage (*P* < 0.01), T stage (*P* < 0.05), N stage (*P* < 0.05), and vital status (*P* < 0.001). The correlations between risk score and AJCC stage, T stage, N stage, M stage, vital status, smoking history, gender, and age were shown in Fig. 7a–h.

**Fig. 8:**
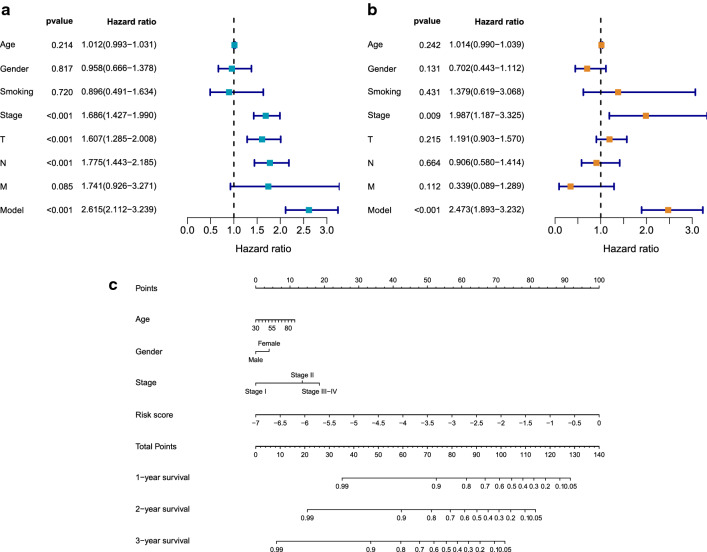
Forest plots and the nomogram for the prognosis of LUAD patients. **a** The forest plot of univariate Cox regression analysis evaluating prognostic effects of clinical features and the risk model for LUAD patients. **b** The forest plot of multivariate Cox regression analysis evaluating prognostic effects of clinical features and the risk model for LUAD patients. **c** The nomogram predicting the overall survival probability of patients with LUAD

The prognostic model and clinical variables including age, gender, smoking history, AJCC stage, T stage, N stage and M stage were sent to univariate and multivariate Cox regression analyses. In the univariate analysis, AJCC stage, T stage, N stage and the risk score model were associated with adverse clinical outcomes (Fig. 8a). Distant metastasis, the widely recognized predictor for bad OS, was not correlated with OS in this analysis, which may be caused by too few samples with M1 stage (N = 20) in this dataset. In multivariate analysis, only AJCC stage and the risk score model were associated with bad clinical outcomes for LUAD patients, indicating their roles as independent prognostic factors (Fig. 8b). A nomogram was then plotted for clinical application (Fig. 8c).

### Construction of splicing network

**Fig. 9:**
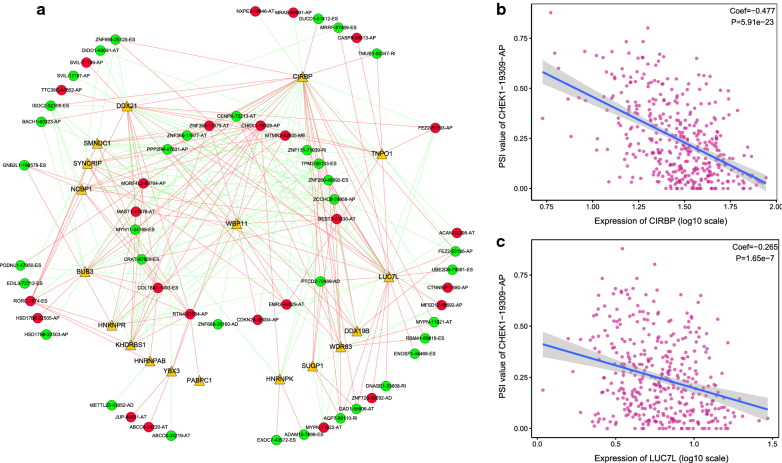
Correlation analysis between splicing factors and ASEs in the LUAD cohort. **a** The splicing network for splicing factors and ASEs. Yellow nodes indicate splicing factors, red nodes indicate poor survival associated ASEs, and green nodes represent good survival associated ASEs; Red lines represent positive correlations, and green lines represent negative correlations. **b** The correlation between PSI values of CHEK1-19309-AP and the expression of *CIRBP*. **c** The correlation between PSI values of CHEK1-19309-AP and the expression of *LUC7L*

We conducted univariate Cox analysis for the 390 SFs and found 18 SFs to have significant effects (*P* < 0.01) on OS of LUAD patients. Spearman test was used to identify correlations between survival-related SFs and ASEs, and a correlation network was established using Cytoscape software (Fig. 9a). The network contains 18 SFs (triangle), 35 protective ASEs (green circle), and 25 risk ASEs (red circle). Proportions of positive regulation (red line) effects and negative regulation (green line) effects were similar in the splicing network. Among these SFs, CIRBP and LUC7L regulated the most ASEs (38 and 29 ASEs, respectively). And CHEK1-19309-AP had correlations with the most SFs (15 SFs). Figure 9b, c shows the correlations between these most representative ASEs and SFs.

## Discussion

Due to the heterogeneity and complexity of cancers, detecting, monitoring, and managing cancers are difficult for clinicians. With the deepening of scientific researches, scientists have unraveled more and more molecular characteristics of cancer initiation and progression. In recent years, many promising biomarkers for the diagnosis and prognosis of LUAD were identified. For example, CAV1 and DCN play critical roles in LUAD cell proliferation inhibition and progression regulation [25], and long noncoding RNA *DGCR5* is an anti-apoptosis marker for LUAD and can promote LUAD progression [26]. Restricted by the sophisticated mechanisms behind LUAD, one single biomarker may only be effective on a proportion of patients. Therefore, many diagnostic or prognostic panels were come up based on various types of biomarkers to make the prediction more applicable and more effective [27–29]. However, most of these studies were restricted to exploration in transcriptome aspect, utilizing mRNAs, long non-coding RNAs, or microRNAs for the construction of a predictive model.

In the last decades, abnormal ASs and presences of specific ASEs have been identified as driven factors for cancers by many studies [30, 31]. For example, the splicing of BCL2L1 pre-mRNA generates two isoforms: the anti-apoptotic isoform Bcl-XL and the pro-apoptotic isoform Bcl-XS. Shifts of BCL2L1’s splicing patterns between those two isoforms can influence the apoptosis of LUAD cells, resulting in the progression or suppression of LUAD [32]. Based on this mechanism, researchers utilized antisense oligonucleotides to push the splicing of BCL2L1 pre-mRNA towards its pro-apoptotic isoform Bcl-XS, which prompted the apoptosis of LUAD A549 cells in vitro [32]. Besides, a previous study reported that the mRNA ratio of Lamin C and Lamin A was increased in all clinical stages of breast cancer and the splicing switch of Lamin A/C alternative splice variants may be of diagnostic use [33]. Apart from participating in chemoresistance pathways, AS can also influence the efficacy of chemotherapeutic agents by the aberrant splicing of molecular targets [34]. In addition to a single AS biomarker, Li et al. and Zhao et al. identified prognostic models for NSCLC using data mining techniques [12, 35]. These studies indicated specific ASEs could be useful tools for the prediction and treatment of LUAD.

However, ASEs implicated in splicing pattern shifts of LUAD or splicing model determining LNM status were rarely explored. The main methods we used in this study for feature selections and classifier constructions are based on random forests. Random forests take advantage of two machine learning methods: bagging and random feature selection. One of the most significant advantages of random forest approaches is their accuracy benefited from the random split of the whole dataset into a training set and a validation set, which contributes to the removal of outliers and noise, resulting in its superior performance over other methods [36]. Based on random forests, Boruta can reduce the influence of random fluctuations and correlations by adding randomness to the dataset and identify features that are really important to the outcome [17]. Besides, another adaption of random forests, RSF model, provides researchers a method to deal with right-censored survival data using decision trees [37]. For these reasons, there is a growing interest in the application of random forest algorithms in bioinformatics fields. To our knowledge, no previous study has used random forest methods or machine learning methods to identify AS signatures in LUAD. Here, by initiatively using several machine learning methods, we integratively analyzed the AS data of LUAD patients and identified a series of AS biomarkers.

In this study, we identified 24 pairs of contrarily expressed ASEs participating in the transitions between risky and protective isoforms for LUAD. Being identical to the splicing of BCL2L1 and Lamin A/C as mentioned above, these biomarkers may have similar therapeutic or diagnostic values for LUAD. Besides, our results also indicate shifts in splicing patterns of QKI, a well-known AS regulator are also strongly correlated with the development of LUAD [38]. The skewed distribution of classes may compromise the result of data mining, so we utilized a data resampling technique to get data with balanced class distribution [39]. While further analysis was focused on the correlations between ASEs, which were not concerned with the distributions of normal and LUAD samples, we used the original imbalanced data for the correlation analysis.

The prognosis of LUAD is significantly correlated with LNM statuses. Previous data displayed 5-year OS of LUAD patients with LNM was 26–35%, while the 5-year OS of LUAD patients without LNM was more than 95% [40]. The 12-ASE-based classifier for LNM showed high sensitivity and specificity in five-fold cross-validation with over 0.7 AUC values in all folds.

We also constructed a prognostic model using 16 ASEs. We first selected survival-related ASEs by the combination of Cox regression and RSF, whose results could be more reliable than utilizing a single method [21]. Then the final list of genes for the prognosis model was selected by forward selection model using R package rbsurv. Based on robust likelihood, this algorithm is widely used for survival model construction [41–43] by utilizing the classical forward selection method to generate a series of models and select an optimal one. Compared with other survival analyses such as artificial neural network-based or deep learning-based survival models, this algorithm is straight-forward and user-friendly in the R programming environment. Although least absolute shrinkage and selection operator (LASSO) Cox regression model is also a popular and automated method for constructing a survival model, the robust partial likelihood-based Cox regression model employed in this study could not only help establish a robust predictive model but also provide the relative importance for survival of each ASE intuitively by calculating mean log-likelihood. This prognostic model was further validated in the internal test set and AUC in 1, 3, 5, and 10 years was 0.766, 0.812, 0.800, and 0.800, respectively, showing the robust predictive capacity. Further study revealed correlations between the risk score model and AJCC stage, T stage, N stage and vital status. These clinical parameters are all OS-relevant and the prognostic model was an independent risk factor. TNM stage is widely used to evaluate the prognosis of LUAD patients. However, the limitation of risk factors of this system makes it impossible to predict OS of LUAD patients precisely. Therefore, we built the nomogram as shown in Fig. 8c to help clinical prediction.

The splicing network built in this study showed the importance of CIRBP and LUC7L as AS regulators. As a stabilizing RNA-binding protein, CIRBP regulates multiple cancers through stabilizing specific mRNAs translating into cancer-associated proteins and modulating inflammation [44]. A recent study also proved its anticancer role in NSCLC [45]. *LUC7L* is rarely studied and encodes a putative RNA-binding protein, contributing to the metastasis of breast cancer [46, 47]. The ASE of *CHEK1* showed the most correlations with SFs. Besides, *CHEK1* encodes the cell cycle checkpoint kinase 1, which is a key kinase for DNA damage response and participates in the cell cycle regulation [48]. Evidence indicated *CHEK1* may implicate with multiple cancers, including NSCLC, breast cancer, and ovarian cancer [49–51]. Our finding suggests its function in cancer progression could be strongly influenced by ASs. In addition, the splicing patterns of most of the biomarkers in the current study are AP, AT, and ES, suggesting the main splicing patterns in LUAD initiation and development.

The limitations of our study should be mentioned too. First, there was a lack of another AS dataset for external validation. Second, the concrete molecular mechanisms of these biomarkers are still unknown because of lacking in vitro or in vivo experiments. In future studies, we will perform in-depth studies to validate our current findings.

## Conclusion

In conclusion, we identified 24 pairs of splicing isoforms strongly correlated with the splicing shifts of LUAD and established two useful AS models to identify LNM and predict OS for LUAD patients. Our findings highlight the importance of AS for LUAD. Biomarkers identified in the present study may provide a new strategy for the diagnosis and treatment of LUAD.

## Supplementary information

**Additional file 1: Table S1.** All samples included in this study.

**Additional file 2: Table S2.** Samples used for differentiating normal and tumor tissues.

**Additional file 3: Table S3.** Samples for recognizing lymph node metastasis (LNM).

**Additional file 4: Table S4.** Information of samples included in the construction of prognostic model.

**Additional file 5: Table S5.** Feature importances and selection results presented by Boruta algorithm for the splicing events differentiating normal and tumor tissues.

**Additional file 6: Table S6.** Feature importances and selection results presented by Boruta algorithm for the classifier recognizing lymph node metastasis (LNM).

**Additional file 7: Table S7.** Unvariate Cox regression results.

**Additional file 8: Table S8.** Survival-related alternative splicing events selected by random survival forest model.

**Additional file 9: Table S9.** Alternative splicing events and their coefficient in the prognostic model.

## Data Availability

The datasets used and analyzed during the current study are available from the corresponding author on reasonable request.

## Abbreviations

AS: Alternative splicing
LUAD: Lung adenocarcinoma
TCGA: The Cancer Genome Atlas
ASEs: Alternative splicing events
LNM: Lymph node metastasis
SFs: Splicing factors
ROC: Receiver operating characteristic
AUC: Area under the receiver operating characteristic curve
NSCLC: Non-small cell lung cancer
OS: Overall survival
RSF: Random survival forest
ES: Exon skip
RI: Retained intron
AD: Alternate donor
AA: Alternate acceptor
AP: Alternate promoter
AT: Alternate terminator
ME: Mutually exclusive exon
SD: Standard deviation
CHF: Cumulative hazard function
AIC: Akaike Information Criterion
KEGG: Kyoto Encyclopedia of Genes and Genomes
